# The pharmaceutical solvent N-methyl-2-pyrollidone (NMP) attenuates inflammation through Krüppel-like factor 2 activation to reduce atherogenesis

**DOI:** 10.1038/s41598-020-68350-2

**Published:** 2020-07-15

**Authors:** Marta Roche-Molina, Bryn Hardwick, Cristina Sanchez-Ramos, David Sanz-Rosa, Dirk Gewert, Francisco M. Cruz, Andres Gonzalez-Guerra, Vicente Andres, Joaquin A. Palma, Borja Ibanez, Grahame Mckenzie, Juan A. Bernal

**Affiliations:** 1 Centro Nacional de Investigaciones Cardiovasculares (CNIC), Melchor Fernandez Almagro 3, CP28029 Madrid, Spain; 2 MRC Cancer Unit At the University of Cambridge, Hutchison/MRC Research Centre, Box 197, Biomedical Campus, Hills Road, Cambridge, CB2 0XZ UK; 3 CIBERCV, Madrid, Spain; 4 Department of Medicine, Universidad Europea de Madrid, Madrid, Spain; 5 DG Bioconsult Ltd, 50 Gilbert Road, Cambridge, CB4 3PE UK; 6 Department of Development, Grupo STIG, Velázquez 11, 28001 Madrid, CP Spain; 7 IIS-Fundación Jiménez Díaz University Hospital, Madrid, Spain

**Keywords:** Atherosclerosis, Drug safety, Transcription, Inflammation

## Abstract

N-methyl-2-pyrrolidone (NMP) is a versatile water-miscible polar aprotic solvent. It is used as a drug solubilizer and penetration enhancer in human and animal, yet its bioactivity properties remain elusive. Here, we report that NMP is a bioactive anti-inflammatory compound well tolerated in vivo, that shows efficacy in reducing disease in a mouse model of atherosclerosis. Mechanistically, NMP increases the expression of the transcription factor *Kruppel-like factor 2 (KLF2)*. Monocytes and endothelial cells treated with NMP express increased levels of *KLF2*, produce less pro-inflammatory cytokines and adhesion molecules. We found that NMP attenuates monocyte adhesion to endothelial cells inflamed with tumor necrosis factor alpha (TNF-α) by reducing expression of adhesion molecules. We further show using *KLF2* shRNA that the inhibitory effect of NMP on endothelial inflammation and subsequent monocyte adhesion is KLF2 dependent. Enhancing KLF2 expression and activity improves endothelial function, controls multiple genes critical for inflammation, and prevents atherosclerosis. Our findings demonstrate a consistent effect of NMP upon KLF2 activation and inflammation, biological processes central to atherogenesis. Our data suggest that inclusion of bioactive solvent NMP in pharmaceutical compositions to treat inflammatory disorders might be beneficial and safe, in particular to treat diseases of the vascular system, such as atherosclerosis.

## Introduction

Atherosclerosis poses a significant burden to healthcare systems throughout the world. Without access to new, inexpensive treatments, atherosclerosis and its associated cardiovascular complications will likely remain a leading cause of morbidity and mortality worldwide^1^. A compelling body of evidence now describes the importance of endothelial dysfunction and inflammation during the development of atherosclerosis^2^, evidence which has driven the pharmaceutical industry to target inflammatory pathways in the quest for novel therapeutic strategies^3–5^. The classical view states that the initial step of inflammation during atherosclerosis depends on the activation of the vascular endothelium^6^. It is now evident that biomechanical forces exerted upon the endothelial layers by blood flow play a significant role in the resolution of inflammatory insults^7,8^. Gene expression studies have demonstrated major differences in the transcriptional profile exerted upon the endothelium by a healthy, laminar blood flow compared to the complex and turbulent flow associated with early sites of atherosclerotic lesions^6,9,10^. These studies have been extensively recapitulated in in vitro studies on endothelial cell monolayers under shear stress conditions^11^. The consistent message coming out from such studies is that a healthy blood flow provokes an endothelial transcriptional program characterized by increased expression of anti-inflammatory, anti-adhesive and anti-thrombotic factors^6,12^. Of the many factors identified, the zinc finger transcription factor *Krüppel-like factor (KLF2)*^13^ has emerged as a master regulator of the atheroprotective transcriptional program in the endothelium^14,15^ and in various myeloid-derived cell types^16,17^. High-shear stress at the endothelial surface induces KLF2, reducing inflammatory activation and antithrombotic properties^18–20^. Crucially, these studies underline the active role of the endothelial cells and leukocytes as key regulators of vascular function to maintain vascular homeostasis^6,21,22^. Modified lipoproteins accumulation in the subendothelial space aggravates the endothelial cell dysfunction and results in the accumulation of macrophages derived from circulating monocytes in the arterial wall^23–25^. Inflammatory activation of macrophages triggers the secretion of inflammatory cytokines, such as tumour necrosis factor-α (TNF-α), which further promotes endothelial maladaptation by activating NF-κB^26,27^. It has been also shown that KLF2 plays a critical role in regulating proinflammatory activation in monocytes^22^. Such studies provide a striking case for KLF2-enhancing therapies as treatment for atherosclerosis, a disease whose etiology is a direct consequence of inflammation and disrupted vascular hemodynamics^3,28^. Such a hypothesis is supported by the observation that the statin class of anti-atherosclerotic drugs function at least partly through their ability to increase *KLF2* expression^28,29^.

*N*-Methyl-2-pyrrolidone (NMP) is a polar aprotic solvent that is frequently used in industrial and pharmaceutical settings^30^. NMP is remarkably effective in maintaining dispersions of different types of nanomaterials^31–33^. Given its safety profile at established doses^30,34^ and its competence to solubilize multiple types of drugs, NMP has become a common solvent component of in situ forming implants^35–37^, for long-term drug delivery^38,39^. NMP acts as a co-solvent and as a complexing agent in different embodiments as it has been reported to increase the solubility and permeability of multiple compounds^36,40–42^. Transcriptional activity of bromodomain-containing proteins is compromised by NMP interaction with the binding pocket of a different bromodomains, what would contribute to its anti-myeloma and immunomodulatory activity^43^.

In this study, we describe an unexpected effect of the FDA-approved solvent NMP upon *KLF2* expression. NMP enhances expression of *KLF2* at the transcriptional level by activating the *KLF2* promoter. Consequently, NMP displays a consistent range of anti-inflammatory effects upon myeloid and endothelial cells in vitro, opposing TNF-α-mediated inflammatory cytokine production and surface adhesion molecule expression. We confirm these observations in an in vivo setting, where NMP inhibits disease progression in a murine model of atherosclerosis. Finally, we demonstrate that these effects also extend to ex vivo human leukocytes, where NMP inhibits leukocyte adhesion to an endothelial layer. As an FDA-approved solvent with a well-established safety profile, NMP may represent a unique opportunity for expedited clinical development for atherosclerosis, especially as a supportive compound to be used in combination with existing therapeutic agents.

## Results

### NMP activates *Klf2* transcription

**Figure 1:**
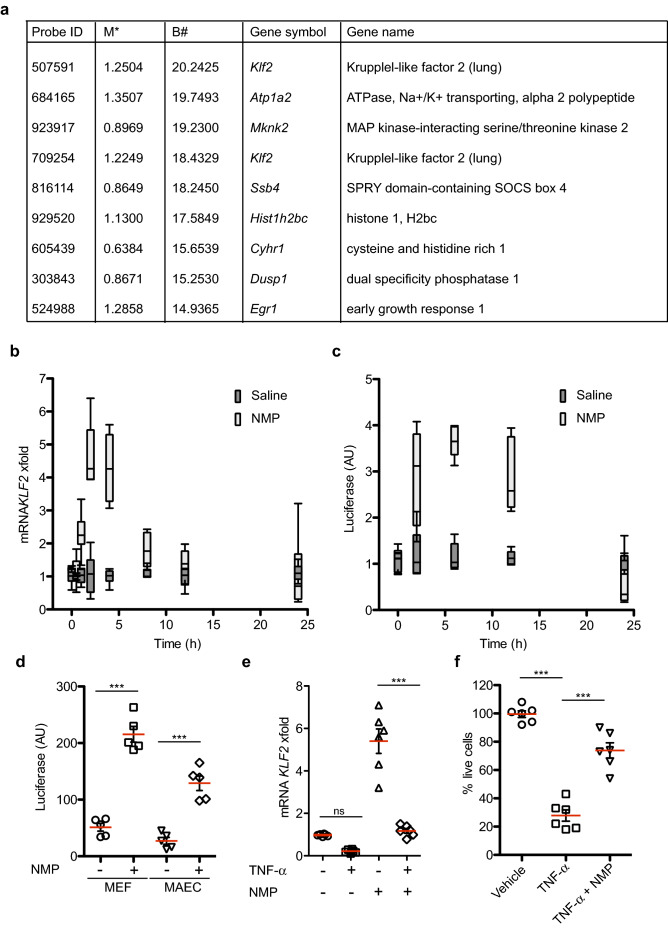
Bioactive NMP is a *Klf2* transcriptional activator. (**a**) Microarray analysis showing top 10 genes up regulated in C2C12 cells by addition of 5 mM NMP for 8 h. (**b**) Real-time PCR analysis of *Klf2* mRNA in mouse C2C12 cells at different time points after NMP administration. *Klf2* mRNA amounts are normalized to *Gapdh* mRNA and are presented relative to the level observed in untreated cells. Data are presented as mean ± SEM; n = 6. (**c**) C2C12 cells transfected with a luciferase Klf2-promoter reporter analyzed at different time points w/wo NMP (1 mM). The luciferase activity was measured and normalized to Renilla activity, and represented in arbitrary units (AU) from two independent assays (n = 6). (**d**) MEF and MAEC cells transfected as in (**c**) were treated with or without NMP. Luciferase assays were performed 24 h after transfection. Data are presented as mean ± SEM; n = 5 *** p < 0.001 (one-way ANOVA with the Bonferroni comparison post-test). (**e**) TNF-α mediated (10 ng/ml) inhibition of *Klf2* mRNA is suppressed by NMP (1 mM). Data are presented as mean ± SEM; n = 6, ns *p* > 0.05, *** *p* < 0.001, one-way ANOVA. (**f**) NMP pretreatment for 6 h protects against cell death induced in C2C12 cells activated with TNF- α for 24 h (n = 6; ***p < 0.001, one-way ANOVA with the Bonferroni comparison post-test).

Previous studies have demonstrated that NMP can affect stem cell differentiation by promoting Bone Morphogenic Protein (BMP)-dependent responses^44^. In order to ascertain the mechanism by which NMP influences these processes, we established a system in the murine C2C12 cell line, which differentiates in response to BMP2^45^. As previously reported, NMP potentiated BMP2-dependent differentiation in this system (data not shown). We further investigated the mechanism by which this might occur by performing transcriptional profiling using mouse genome survey microarrays. A cursory analysis of the results revealed an effect of NMP on the expression of *Klf2*. In these microarray experiments, NMP enhanced expression of *Klf2* after 8 h of treatment (Fig. 1a). This observation was confirmed by Q-PCR analysis from independent experiments (Fig. 1b). The importance of *Klf2* in atheroprotection^14^ led us to investigate this serendipitous effect further.

Reasoning that this increase in *Klf2* might be due to transcriptional activation, we investigated whether NMP could affect the activity of the *Klf2* promoter. To address this question, we cloned the mouse *Klf2* promoter upstream of a firefly luciferase reporter with an upstream adenoviral TATA box. Transient transfection of this construct into C2C12 cells showed that the putative *Klf2* promoter drove a significant luciferase activity over and above that of the vector lacking a promoter, confirming its identity as regulatory element. NMP treatment was able to transactivate the *Klf2* promoter construct in a time-dependent manner (Fig. 1c), confirming the microarray and Q-PCR data. Furthermore, this transactivation activity of NMP over the *Klf2* promoter was not cell type specific, as we could observe activation in mouse embryonic fibroblast (MEF) and mouse aortic endothelial cells (MAEC) (Fig. 1d).

We asked whether NMP's effect on *Klf2* transcription has a functional effect downstream. Pro-inflammatory cytokines have been reported to down-regulate *Klf2* expression^46^. We confirmed this in C2C12 cells: TNF-α treatment decreased *Klf2* mRNA levels as measured by Q-PCR, but this effect was overcome in the presence of NMP, leading to the maintenance of *Klf2* mRNA levels (Fig. 1e). This ability of NMP to oppose TNF-α-mediated reduction of *Klf2* mRNA was correlated with increased cell viability (Fig. 1f), where NMP treatment markedly inhibited the ability of TNF-α to kill C2C12 cells.

**Figure 2:**
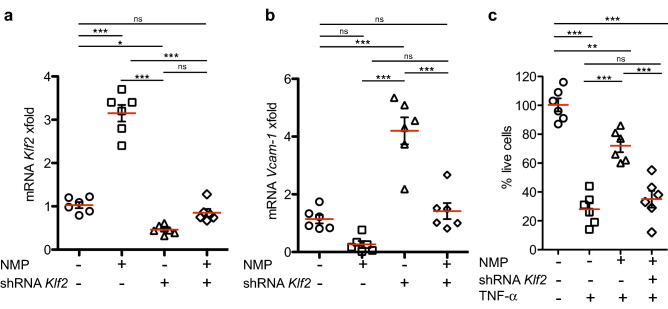
NMP ameliorates TNF-α-induced-apoptosis through the Klf2 pathway. (**a**) Exponentially growing non-synchronized cells were transduced with lentiviral particles encoding for shRNA against *KLF2* mRNA (“shRNA”) or for a non-specific shRNA as control; 72 h after infection cells stimulated with TNF- α for 6 h were exposed to NMP (1 mM), RNA was purified and *Klf2* (**a**) and *Vcam-1* (**b**) mRNA levels were analyzed by QPCR, and (**c**) cell viability was measured by flow cytometry. The results in C2C12 cell line show at least two independent biological replicates (n = 6). Error bars represent the standard error of the means (SEM). Significant differences are represented by asterisks: *p < 0.05, **p < 0.01 and ***p < 0.001, or non-significant (ns), by one-way ANOVA with Bonferroni’s multiple comparison test.

To determine whether *Klf2* regulates the transcriptional program that drives survival of C2C12 cells after TNF-α administration, we used lentiviral shRNA construct to down-regulate *Klf2*. QPCR analysis confirmed that *Klf2*-specific shRNA effectively suppressed *Klf2* expression in cells, whereas a control shRNA had no effect (Fig. 2a). We determined that shRNA-mediated *Klf2*-knockdown effectively suppresses transcription of target genes like *Vcam-1* (Fig. 2b), and found that NMP protective effect after TNF-α treatment on survival of C2C12 is *Klf2*-specific. *Klf2* shRNA knockdown substantially reduced the cell survival after co-administration of TNF-α and NMP, whereas the control shRNA had no significant effect (Fig. 2c). These data strongly suggest that NMP treatment provokes effects which oppose pro-inflammatory responses and cell survival in cell-based assays.

### NMP is tolerated and increases *Klf2* expression in vivo

**Figure 3:**
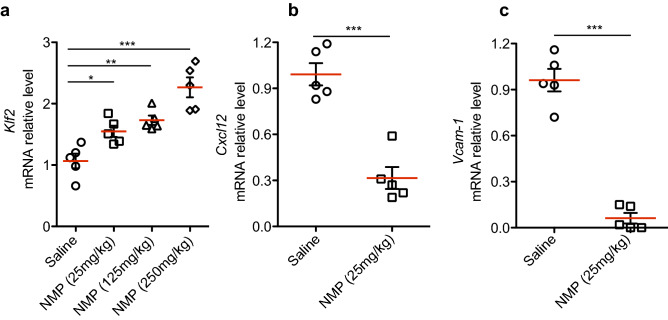
NMP is bioactive in vivo. (**a**) Real-time PCR analysis in liver samples of mRNA expression of (**a**) *Klf2* mRNA in mice treated with 25, 125 and 250 mg/kg of NMP. ***p < 0.001, **p < 0.005,*p < 0.01 by one-way ANOVA with Bonferroni’s multiple comparison test (n = 5 mice). Each data point denotes a liver sample from an individual mouse. (**b,c**) endogenous mouse *Cxcl12* and *Vcam-1* mRNA in treated mice with 25 mg/kg mouse. mRNA amounts are normalized to *Gapdh* mRNA relative to the level of liver samples in non-treated animals. (n = 5; ***p < 0.001, unpaired Student’s t test).

We reasoned that NMP's status as an FDA-approved pharmaceutical excipient might make it an attractive solvent with intrinsic therapeutic activities for treating atherosclerosis. Given the in vitro effects of NMP described above, combined with its favorable safety profile, we tested its in vivo efficacy. We first confirmed previous safety assessments of NMP^34,47^. Adult C57BL6/J mice (8–12 weeks old) were injected *i.v*. or *i.p*. with NMP at 25, 125 and 250 mg/kg, and sacrificed twenty four hours after treatment. Histopathology was performed and analyzed for macroscopic alterations in liver, heart, kidney, spleen, gut, skeletal muscle, and lung to study the effects of NMP: no inflammation, fibrosis or necrosis were seen at any doses. In addition, serum levels of alanine aminotransferase (ALT) were evaluated as a marker for potential metabolic hepatocellular injury. We observed no differences in ALT levels between NMP- and saline-treated mice (Supplementary Fig. 1a). To confirm a transcriptional effect of NMP in vivo, we administered NMP at 25, 125 and 250 mg/kg intravenously and prepared total RNA from liver 24 h post-treatment. *Klf2* mRNA levels after NMP administration were increased in a dose-dependent manner compared with saline-treated animals (Fig. 3a). In addition to *Klf2*, we noted that NMP treatment caused a decrease in *Cxcl12* (Fig. 3b) and *Vcam-1* (Fig. 3c) mRNA expression. The *Cxcl12* promoter region has previously been shown to be repressed by KLF2^48^.

Since mouse models of atherosclerosis develop over a prolonged period, we also tested the tolerability of long-term NMP treatment based on previous work^47^. NMP was administered in drinking water at 0.75 mg/ml (150 mg/kg/day)^47^ to mice fed a high-cholesterol diet for a 12-week period. No evidence for differential hepatocellular damage between the groups was observed (Supplementary Fig. 1b). Taken together these data demonstrate that NMP can enhance *Klf2* expression and its associated downstream pathways in vivo.

### NMP treatment increases atheroprotection in HFD-fed *ApoE*^−/−^ mice

**Figure 4:**
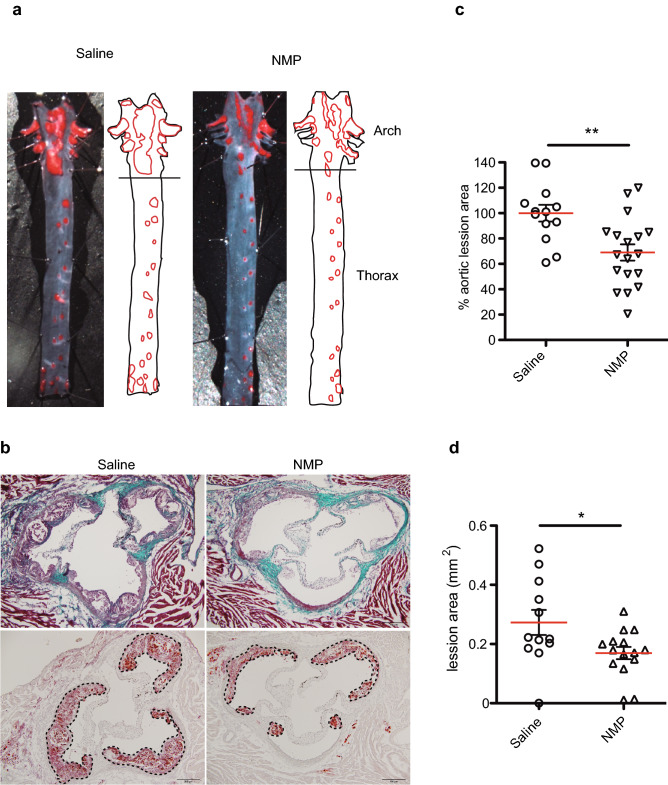
NMP attenuates atherosclerosis development in ApoE − / − mice. (**a**) Representative *en face* Oil Red O staining of aortas from treated and untreated NMP mice maintained on HFD for 12 weeks. (**b**) Representative Masson’s trichrome (upper panel) and Oil Red O (lower panel) staining in aortic root sections from HFD-fed NMP treated and untreated mice. Scale bar, 200 μm. (**c**) Quantitative analysis of atherosclerotic lesion size in Oil Red O-stained in aortic arch region from (**a**). (**d**) Quantitative analysis of atherosclerotic lesion size in Oil Red O-stained aortic sections from (**c**). (n = 12–18; *p < 0.01, **p < 0.005, unpaired Student’s t test).

Having shown that NMP exerts an anti-inflammatory effect in cultured cells and that it is also well tolerated and active in vivo, we tested NMP in an animal model of atherosclerosis. The hypercholesterolemic *apolipoprotein-E*-deficient (*ApoE*^*−/−*^) mouse, when fed with a high fat diet (HFD) is used as a standard model of atherosclerosis^49^. To assess atherosclerotic plaque development after long-term NMP treatment, HFD-fed mice were allowed access to either untreated drinking water, or drinking water containing 0.75 mg/ml NMP for a period of 12 weeks. At the end of this treatment period, mice were euthanized and their aortas analyzed for signs of atherosclerosis. Histological analysis of *en face*-prepared aortas stained with Oil Red O (Fig. 4a) demonstrated a reduction in atherosclerotic area in aortic arch of NMP-treated mice (Fig. 4b). As shown by Oil Red O, NMP treatment led to a decrease in the size of atherosclerotic plaques in the aortic arches of HFD-fed *ApoE*^*−/−*^ mice. Quantitative image analysis of the total area of atherosclerotic lesions (Fig. 4c) showed a significant effect of NMP treatment. Further histological analysis at the aortic sinus also demonstrated that NMP treatment had reduced the mean aortic root lesion area compared with untreated HFD-fed *ApoE*^*−/−*^ mice (Fig. 4d).

**Figure 5:**
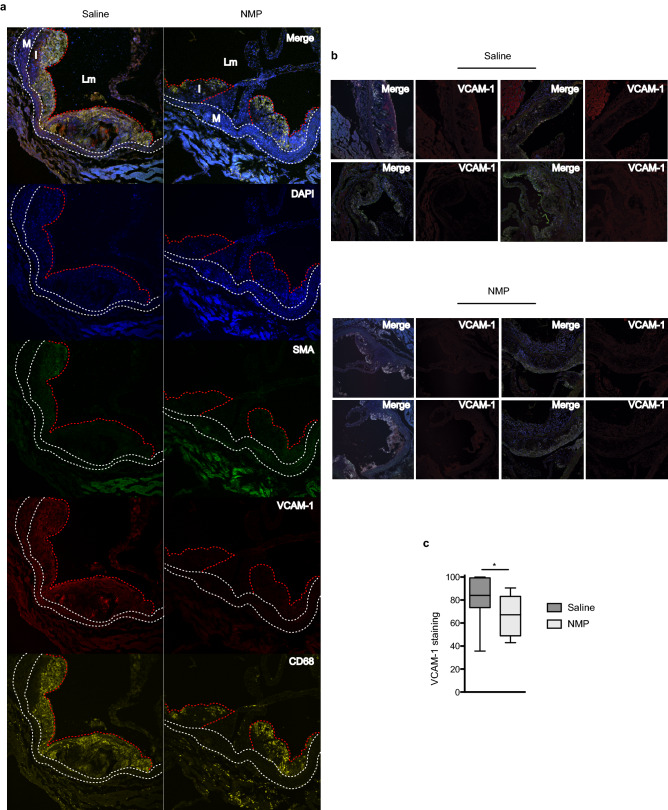
NMP treatment reduces macrophages infiltration and inflammation. (**a**) Representative immunostaining of macrophages, smooth muscle cells (SMC) and activated endothelium in aortic sinus lesions of NMP treated and untreated C57BL/6 J mice fed with HFD for 12 weeks. Lesions were stained for biomarkers of macrophages (CD68; yellow), SMCs (α-actinin; green) and stressed endothelium (Vcam-1; red); nuclei were stained with DAPI (blue). Merged images are also shown. Lm, aortic lumen, M, tunica media, and I, tunica intima. White dashed lines demarcate the elastic lamina. Bars, 200 μm. (**b**) Vcam-1 and merge staining as in (**a**) from different animals. (**c**) Quantitative analysis of relative Vcam-1 immunostaining relative to the aortic sections size from (**b**).

To characterize the effects of NMP on atherosclerotic lesions, we stained aortas for various inflammatory markers and analyzed their expression by immunofluorescence (Fig. 5a). Vcam-1 staining was markedly reduced with NMP treatment, and, consistent with this, macrophage infiltration into sites of atherosclerosis, as assessed with CD68 staining, was also significantly reduced. Our staining also showed that NMP administration inhibited Vcam-1 induction (Fig. 5b,c) in the alpha-actin-2 (α-SMA) smooth muscle cells^50^ present at the atherosclerotic lesions. These results confirmed that NMP treatment has a striking inhibitory effect upon the inflammatory status of the aorta during atherogenesis.

### NMP attenuates expression of inflammation genes in the aorta

**Figure 6:**
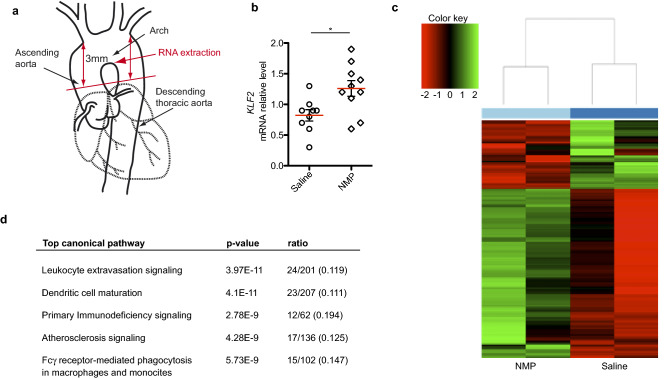
Gene expression signature of NMP treatment in the atherosclerotic aorta. (**a**) The diagram shows the aortic section plane (red line) and the aortic tissue sampled for RNA-seq studies. (**b**) Real-time PCR analysis of *Klf2* mRNA in aortic arches after NMP or saline administration. Klf2 mRNA amounts are normalized to *Gapdh* mRNA and are presented relative to the level observed in untreated aortas. (**c**) Heat map of top genes differentially expressed in aortic arches between NMP and saline treated mice. Scaled expression values are color-coded according to the legend on the left. The dendrogram depicts hierarchical clustering based on the top differentially expressed genes. The top bar indicates the treatment: light blue, NMP; blue, saline control. (**d**) Top canonical pathways that were significantly deregulated by NMP treatment, as identified by Ingenuity Pathway Analysis. The significance of association between altered genes and the canonical pathway was assessed using a right-tailed Fisher's exact test to calculate a *p* value determining the probability that the association is explained by chance alone. Ratios defining the proportion of deregulating genes from a pathway related to the total number of molecules that make up that particular pathway are also indicated.

*Klf2* expression in this location plays a key atheroprotective role, preventing local inflammation and the subsequent formation of atherosclerotic lesions. We therefore tested whether NMP administration could influence gene expression in the aortic arch. To this end, *ApoE*^*−/−*^ mice were fed with HFD and treated with or without NMP, which was formulated in the drinking water (0.75 mg/ml). Mice were sacrificed after 12 weeks and total RNA was extracted from the aortic arch (Fig. 6a) to study the transcriptome of the aortic arches in these mice. *Klf2* mRNA analysis revealed an induction of expression in aortas of NMP-treated mice (Fig. 6b). In a pilot experiment, we next compared the global expression profiles of *ApoE*^*−/−*^ controls with *ApoE*^*−/−*^ mice treated with NMP (n = 2 for each groups) using next-generation sequencing (NGS). We performed hierarchical clustering on 1 logarithmic fold change of the significant altered genes (*p* < 0.05) between the two experimental groups. The resulting dendrogram showed that co-segregation occurred entirely according to sample type, with a clear division between control- and NMP-treated mice (Fig. 6c). Given the strong consistency and reduced variability between duplicated samples we were able to perform an unsupervised transcriptomic assessment to detect differential alterations in a total of 448 genes, using a cut-off of twofold (Supplementary Table 2). Unbiased Ingenuity Pathway Analysis (IPA) of the molecular and cellular functions of these differentially expressed genes (threshold *p* < 0.05) identified 5 major networks that were differentially affected by NMP treatment (Fig. 6d). The most significant alterations in functional networks were related to genes involved in the immune response, specifically leukocyte adhesion and extravasation. The list of down-regulated transcripts included a striking number of pro-inflammatory factors and adhesion molecules such as *CD5L*, *Ptgs2* (also known as *Cox-2*), *Vcam-1*, *Sele*, *Psgl-1*, *Selplg*, several integrins (*Itgal, Itgax, Itga6, Itgb2*)^51^ and chemokine receptors (*Ccr2, Ccr5*)^52^, many of which are known to play a key role in leukocyte adhesion to the endothelium. Other genes involved in vascular homeostasis and tone (*End-1* and *Nos2*), blood clot dissolution (*Serpine-1*) and lipid metabolism (*Olr1*) were also down-regulated. There were also up-regulated transcripts of interest, notably *Apoa2*^53^, which controls reverse cholesterol transport, and *Ptx3*^54^ a regulator of monocyte differentiation and trafficking. This initial unbiased transcriptomic approach suggests that NMP exerts a dramatic anti-inflammatory effect upon the aortic arch of atherosclerotic mice.

### NMP is active in human monocytes and endothelial cells

**Figure 7:**
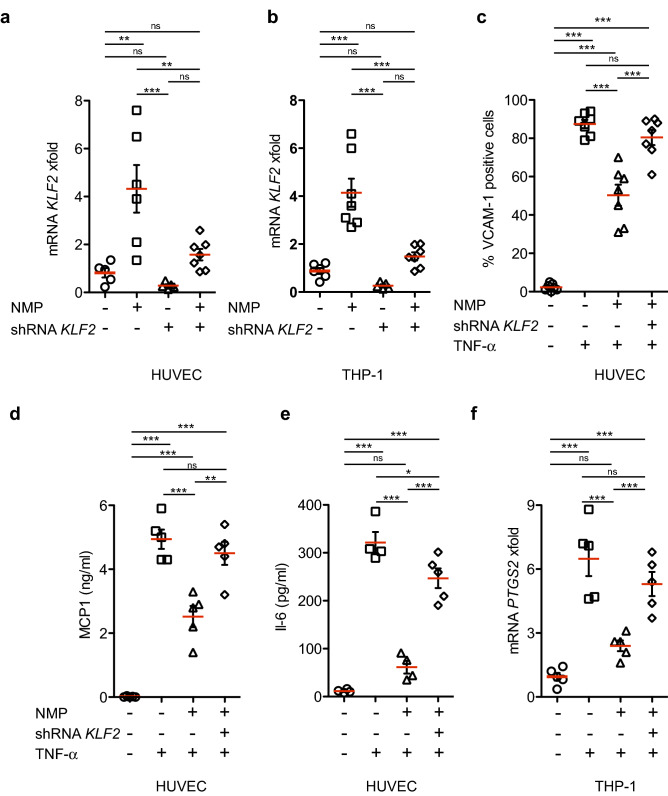
NMP inhibits TNF-α-dependent vascular inflammation by KLF2. (**a**) Real-time PCR analysis of *KLF2* mRNA in human HUVEC and (**b**) THP-1 cells after 24 h of NMP administration. *KLF2* mRNA amounts are normalized to *GADPH* mRNA and are presented relative to the level observed in untreated cells. (**c**) Percentage of VCAM-1 positive HUVEC cells. NMP reduces cytokine release in HUVEC cells after TNF-α stimulation. Supernatants from HUVEC cells were harvested after 24 h of treatment, and the concentration of MCP-1 (**c**) and IL-6 (**d**) measured by ELISA, TNF-α treated with NMP compared with TNF- α untreated cells. (**f**) Real-time PCR analysis of *PTGS2* mRNA in human THP-1 cells. All data is shown as mean ± SEM (n = 4–7). *p < 0.01, **p < 0.005, ***p < 0.001 by one-way ANOVA with Bonferroni’s multiple comparison test.

Our in vivo data in an established murine model of atherosclerosis drove our study towards human cells. For these experiments, we utilized human THP‐1^55^ monocyte and umbilical vein endothelial cells (HUVECs), which are widely accepted as an appropriate proxy for their in vivo counterparts in the human vasculature^56^. We treated THP‐1 and HUVECs with NMP and measured *KLF2* transcript by Q-PCR. As seen in murine cells and in vivo, NMP caused an increase in *KLF2* messenger levels in HUVEC and TPH-1 cells that peaked 4 h after treatment (Fig. 7a,b). We then attempted to correlate this increase in *KLF2* with downstream functional effects. High levels of *KLF2* expression in HUVECs have been previously associated with a state of functional quiescence, characterized by non-responsiveness to inflammatory stimuli such as IL-1 and TNF-α^57^. We therefore hypothesized that NMP would oppose the effects of inflammatory insults to monocytes and endothelial cells. THP‐1 monocyte and HUVECs cells were pre-incubated for 24 h with stealth siRNA targeting human *KLF2* specific siRNA or scramble control before treating them with the inflammatory cytokine TNF-α. To test whether NMP activity on TNF-α is KLF2-dependent, activated cells with or without KLF2 were incubated at the same time with NMP. After additional 24 h, cellular response was assessed by two parameters in HUVEC cells: expression of the surface marker VCAM-1, and production of the pro-inflammatory cytokines MCP-1/CCL2 and IL-6, and *PTGS2* in THP-1 monocytes. As shown in Fig. 7c,d,e,f respectively, NMP had a profound effect upon VCAM-1 surface expression MCP-1/CCL2 and IL-6 secretion, dramatically opposing the effect of TNF-α. Equivalently, we observed an inhibition of *PTGS2* in cultured monocytes incubated with NMP. However, THP‐1 monocyte and HUVECs cells treated with *KLF2*-directed siRNAs partially lost the effect of NMP on TNFα‐induced response. These results correlate with the loss of activation of known KLF2 targets like VCAM-1 in HUVEC cells, and PTGS2 in THP-1 monocytes. These data demonstrate that anti-inflammatory effect of NMP is KLF2-dependent. Furthermore, NMP is not effective only upon mouse cells but indeed has a robust protective effect upon human monocytes and vascular endothelial cells.

### NMP prevents monocyte adhesion

Monocyte adhesion and extravasation from the circulatory system into sites of endothelial cell inflammation is central to atherogenesis. We therefore hypothesized that VCAM-1 down-regulation induced by NMP could be critical to reduce monocyte recruitment and firm adhesion to a dysfunctional human endothelial layer. To analyze a potential VCAM-1 regulated interaction of endothelial cells and monocytes/macrophages, we first performed static adhesion assays using endothelial cells and the THP-1 monocyte cell line. Both control and *KLF2* siRNA treated HUVECs were stimulated with soluble TNF-α and treated or not with NMP. We found that stimulation of HUVECs with TNF-α for 6 h significantly enhances subsequent adhesion of THP-1 cells (Supplementary Fig. 2), that it is diminished by NMP treatment in a KLF2-dependent manner. In contrast, *KLF2* knockdown alter THP-1 cell adhesion compared to HUVECs treated with scramble control. The extent of enhancement of THP-1 cell adhesion to HUVECs induced by *KLF2* siRNA is comparable to that of TNF-α. Increased monocyte adhesion to endothelial cells induced by TNF-α is known to be important during the initiation of an atherosclerotic lesion.

**Figure 8:**
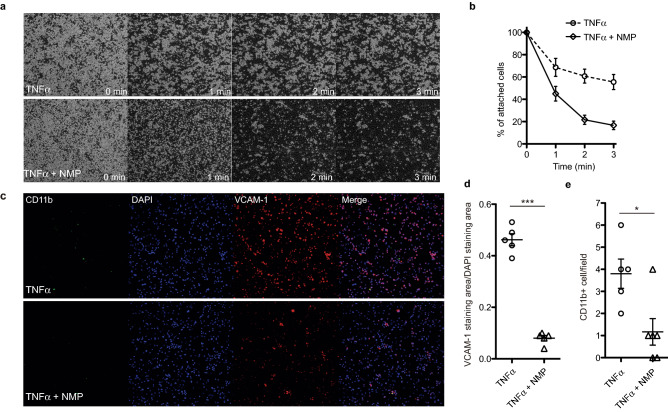
NMP attenuates THP‐1 monocyte adhesion to HUVECs. (**a**) Representative frames (from Supplemental Video 1) showing THP-1 cells adherence to a monolayer of TNF-α pre-activated HUVECs after treatment with NMP or saline. Numbers indicate elapsed time in minutes. (**b**) Quantitative analysis of the percentage of remaining attached THP-1 cells to the HUVEC monolayer by comparison of the percentage of attached cells at the beginning of the flow (time = 0 min). (**c**) Representative immunostaining of attached PBMC to a TNF-α activated HUVEC cells monolaye after 5 min of flow (5 dyn/cm2). PBMCs were stained with CD11b in green, and stressed endothelium with VCAM-1 (red), nuclei were stained with DAPI (blue). Merged images are also shown. (**d**) Quantitative analysis of VCAM-1/DAPI positive staining area from (**c**). Quantitative analysis of CD11b positive stained cells per field from (C). (n = 5; *p < 0.01, ***p < 0.001, unpaired Student’s t test).

To test the ability in a more dynamic scenario we established an in vitro shear stress flow system to test leukocyte adhesion to an endothelial cell layer measured under flow conditions^58^. In the first of these experiments, HUVEC cells were cultured upon µ-Slide VI^0.4^ flow chambers, and then the human monocytic cell line THP-1 was added to this monolayer in media containing either TNF-α alone, or TNF-α plus NMP. After 6 h of incubation, re-circulating flow was applied to the flow chamber at 5 dyn/cm^2^, and video images were captured for 3 min. The results of this experiment are shown in Supplementary Movies 1 and 2, and snapshots taken at different time points from these videos are shown in Fig. 8a. THP-1 cells clearly remain adhered to the HUVEC monolayer in the presence of TNF-alpha alone, including NMP in the media minimizes THP-1 adhesion, and the cells are clearly washed from the HUVEC monolayer with flow. These observations are quantified in Fig. 8b. NMP therefore prevents a model monocyte cell line from adhering to activated human endothelial cells.

To extend this experiment, we once again employed a flow system, but this time used peripheral blood mononuclear cells (PBMCs). HUVECs were plated in µ-Slide VI^0.4^ flow chambers and then treated for 6 h with media containing either TNF-α alone, or TNF-α plus NMP to activate the endothelium. Freshly-isolated PBMCs were then applied to the flow chamber with re-circulation. After 10 min of 5 dyn/cm^2^ of flow, the slides were fixed for immunofluorescent analysis. As shown in (Fig. 8c), NMP treatment inhibited TNF-α-dependent *VCAM-1* expression (quantified in Fig. 8d) and, crucially, inhibited the number of leukocytes (as measured with the pan-leukocyte marker CD11b) able to adhere to the activated endothelial layer (Fig. 8e). Taken together, these data demonstrate that, under our model flow assay, NMP opposes the effects of inflammation upon leukocyte retention and binding to endothelial cell layers, a process which is a key event in the establishment of initial atherosclerotic lesions.

## Discussion

NMP is a common solvent with extensive industrial uses. Its superior solvent properties, combined with its favorable in vivo tolerability and safety profile, have led to its use as a pharmaceutical excipient in a wide range of current therapies^30^. Most notably, it is used in the formulation for a number of clinical oncology compounds^43^. Long assumed to be inert, recent data has shown that NMP exerts dramatic effects in a murine model of multiple myeloma, possibly through its ability to function as an acetyl-lysine mimetic, inhibiting bromodomain-containing proteins such as BRD4^43^. We noticed that some of the anti-inflammatory effects documented in that study are consistent with those we describe here.

Based upon our initial observations that NMP treatment enhanced *Klf2* expression in a murine model of mesenchymal differentiation, our data shows that NMP exerts a dramatic and consistent set of anti-inflammatory effects across a broad range of cell culture, in vivo and ex vivo studies. A major translational finding of this study is our demonstration that NMP halts atherosclerotic progression in the *ApoE*^*−/−*^ mouse model. This mouse, when fed with a high-fat diet, is the classical in vivo model used to study the early stages of atherogenesis. Since our data suggested that NMP influences the molecular and cellular processes involved in the initial inflammation and subsequent extra-vasation required for initiating atherogenesis, we reasoned that this would be an ideal in vivo model. Crucially, the *ApoE*^*−/−*^ model has been used to demonstrate potential clinical efficacy for a number of current atherosclerosis treatments^49^.

Recent advances in our understanding of the molecular basis of atherosclerosis offer new opportunities for therapeutic intervention. The concept that endothelial dysfunction both initiates and then promotes development of atherosclerotic lesions is now well accepted. Therapeutic approaches targeting endothelial and pro-inflammatory processes may therefore prevent the initiation, and indeed the progression, of the atherogenic process by promoting a return to vascular homeostasis. Central to the study presented here is the identification and establishment of KLF2 as a master regulator of the anti-inflammatory response^12,59^. Although initial observation of Klf2 activation by NMP was shown in skeletal myoblasts C2C12 cell line, we also demonstrated that endothelial cells (MAEC and HUVEC cells) response in a similar way. This crucial body of evidence linking KLF2 expression to endothelial health and inflammation has led us to investigate the potential use of NMP as an anti-atherosclerotic agent.

Even though our initial transcriptomic approach uses only 2 mice per group, the consistency and the minor variability observed between samples set up the basis to demonstrate that NMP is able to activate KLF2 transcriptional activity to blocks monocyte adhesion to endothelial cells and to reduce atherosclerosis development. Based on our *in vitro* and *in vivo* results, we show that NMP inhibits TNFα‐stimulated upregulation of VCAM1 what end up in reducing monocyte adhesion to endothelial cells. As VCAM1 is a well‐known transcriptional target of KLF2, we investigated whether the downregulation of VCAM1 by NMP is KLF2-dependent^15^. In fact, KLF2 overexpression inhibits TNFα‐mediated induction of the proinflammatory molecule, such as VCAM1, in endothelial cells, what further supports that the mechanism for the antiatherosclerotic effect of NMP is at least partially attributed to KLF2‐dependent anti‐inflammatory activities. The consequent antiatherosclerotic effects accompanying KLF2 upregulation by NMP may include promoting a set of genes with demonstrated anti‐thrombotic, ‐inflammatory, and ‐proliferative functions. It is important to acknowledge that atherosclerosis is a complex chronic inflammatory disease which development depends on the functional interplay among multiple cell types^60^. Given the fact that KLF2 is expressed in several cell types involved in atheroma plaque development, including monocyte/macrophage, endothelial cells, smooth muscle cells, and T cells,^16,61,62^ it is plausible that the atheroprotective effects of NMP is the result of that combined activity. We demonstrate in the *ApoE*^−/−^ mouse model that NMP affects the inflammatory response of these cell types during the formation of the atherosclerotic lesion. Therefore, the precise contribution of distinct cell types in NMP‐mediated atheroprotection justifies future investigations.

The data presented here clearly demonstrates that NMP can halt atherosclerotic progression. We propose two possible translational routes for exploiting this finding. Firstly, NMP is a solvent which could be incorporated into many polymer-based biomaterials. One could therefore envisage its use in a drug-eluting stent, which might allow local delivery of NMP to the area of atherosclerotic plaques. Drug-eluting stents have been used extensively, and with great success, in combination with anti-proliferative drugs such as paclitaxel^63^ and sirolimus^64^ in order to prevent post-surgical restenosis. Given the effect of NMP on KLF2 levels, combining NMP with such compounds may provide an additional benefit by promoting a transcriptional profile associated with a healthy, non-inflamed endothelial and leukocytes.

Secondly, NMP has a well described safety and tolerability profile in the clinic as a solvent, and, therefore, might conceivably be considered for use in the formulation of statins, which are currently the favored medicine to lower plasma low-density lipoprotein (LDL) concentrations and to treat atherosclerosis. However, despite being fairly well tolerated, statins are associated with muscular symptoms, ranging from mild myalgia to rhabdomyolysis. These side effects occur in up to 20% of statin users, and are a leading cause of non-compliance. Our data present the intriguing possibility of formulating statins with NMP to gain additional anti-atherosclerotic potential from this active solvent. In addition, it is possible that statin dose might be reduced if used in conjunction with NMP, thereby likely reducing side-effects and subsequent non-compliance. The data presented here clearly indicate that NMP warrants further investigation into its use to complement statin administration for the treatment of atherosclerosis.

## Materials and methods

### Animals

All experiments were carried out in accordance with the CNIC Institutional Ethics Committee recommendations, and were approved by the Animal Experimentation Committee of Comunidad de Madrid project number PROEX 019/17. Atherosclerosis mouse model and wild type control were maintained as previously described^65^ with free access to food and water. Wild-type mice and homozygous *ApoE*-deficient mice (*ApoE*^*−/−*^), both on the C57BL/6 J genetic background were obtained from Charles River Laboratories. Mice were fed a low-fat standard rodent diet (Teklad global rat/mouse chow, Harlan Interfauna). When indicated, mice were switched to a high-fat diet containing 0.75% cholesterol (Ssniff, S8492-E010) and analyzed for atheroma plaque formation after 84 days. The mean daily safe^47^ intake of NMP over the 12 weeks period was 150 mg/kg/day administered in drinking water at 0.75 mg/ml. Mean daily water intakes in C57BL/6 J strain ranges from 6-8 ml/day^66^ in 30 g body weight animals.

### Microarray analysis

The murine myoblast C2C12 cell line (ATCC CRL-1772) was cultured in DMEM (Life Technologies 31966-021) supplemented with 10% Fetal Bovine Serum (Life Technologies 10270-106). Total RNA was extracted from cultured C2C12 cells using the RNeasy Mini Kit (Qiagen 74104) as directed by the manufacturer’s instructions. Microarray analysis on the murine C2C12 cell line was carried out by Geneservice Ltd (now Source Bioscience Ltd) using the AB1700 Mouse Genome Survey Array (Applied Biosystems). Analysis was carried out on transcripts after 8 h of treatment with media only (untreated) or 5 mM NMP (Scharlau, ME0498). RNA amplification, labeling, hybridization, and detection were performed following the protocols supplied by Applied Biosystems together with the corresponding kits. The labeled RNAs were hybridized and detected according to the supplied protocols. All expression data was pre-processed and normalized. Differentially Expressed Genes were identified from normalized data.

### Gene knockdown assay and quantitative RT-PCR

For stable knockdown of *Klf2* gene cells were infected with shRNAs or *EGFP* control lentivirus in the presence of polybrene (8 μg ml^−1^). Lentiviral vector pGIPZ (Open Biosystems) was used to knockdown *Klf2* (V3LMM_473116) expression, as well as a Non-silencing lentiviral shRNA was used as a control (NS; RHS4346). A multiplicity of infection (MOI) of 5 for 8 h was used for achieving over 95% transduction (GFP-positive) 72 h after infection. To target human *KLF2* gene Stealth siRNA (HSS145585) was purchased from Life Technologies. Total RNA was extracted and reverse-transcribed from cultured C2C12, THP-1, or HUVECs (Life Technologies, C-003-5C) with the RNeasy Mini Kit (Qiagen 74104) and SuperScript II cDNA synthesis kit (Life Technologies, 18064-014) according to the manufacturer’s instructions. RNA messenger was amplified on a Chromo4 RT-PCR System (BioRad) using the QuantiTect SYBR Green RT-PCR Kit (Qiagen 204243) following the manufacturer’s protocols. Primer sequences are listed in Supplementary Table 1. Expression was calculated comparing threshold cycle (CT) method and normalizing to *Gapdh* (mouse) or *GAPDH* (human) gene expression.

### *Klf2* Luciferase reporter assay

A 460 bp DNA fragment directly upstream of the *Klf2* gene was amplified from mouse genomic DNA using the following primer sequences: Forward, 5′ GCCCCAAACTTCATCCTTCTTGTC 3′; Reverse, 5′ GCCTTATAGGCGCGGCAGGCACAG 3′. The resulting PCR product was cloned into the Firefly luciferase reporter vector pGL3Basic (Promega, E1751). The Klf2 reporter construct was transfected into C2C12 cells with the pRL-TK renilla luciferase normalization vector (Promega, E2241) using Lipofectamine 2000 (Life Technologies 11668019) using supplied protocols. 24 h after transfection, luciferase levels were measured using the Dual-Glo Luciferase Assay System (Promega, E2940) as per manufacturer’s instructions using a BMG Labtech FluoStar plate reader. Firefly luciferase reporter data was normalized to the Renilla luciferase from the same well.

### Apoptosis assay

Apoptosis in C2C12 myoblasts was assessed using the annexin V-FITC apoptosis detection kit (Sigma, Aldrich) in a 6-well plate format according to the manufacturer's instruction. NMP activity was tested in the presence or absence of TNF-α (10 ng/ml) for 24 h. Cells were harvested, washed twice with cold PBS, and resuspended in annexin-V binding buffer at 1 × 10^5^ cells/ml. A 250 μl aliquot of the mix was incubated with equal amounts (5 μl) of annexin-V and propidium iodide (PI, 20 μg/ml) in the dark at room temperature for 15 min, before flow cytometer (BD Biosciences, USA) analysis. PI and annexin-V positive cells were considered as control live cells. Cells positive for annexin-V, negative or positive for PI were considered apoptotic, whereas cells only PI positive were considered necrotic. The percent of apoptotic cells was calculated as the annexin-V positive cells relative to the overall number of cells.

### cDNA library preparation and next generation sequencing (NGS)

Samples were prepared and analyzed as previously described^67^. Total RNA was quantified and purity checked using a NanoDrop ND-1000 (Thermo Scientific). RNA integrity was verified using an Agilent 2100 Bioanalyzer (Agilent Technologies). 500 ng of total RNA were used with the TruSeq RNA Sample Preparation v2 Kit (Illumina, San Diego, CA) to construct index-tagged cDNA libraries. Libraries were quantified using a Quant-iT dsDNA HS assay with the Q-bit fluorometer (Life Technologies). Average library size and the size distribution were determined using a DNA 1000 assay in an Agilent 2100 Bioanalyzer. Libraries were diluted to 10 nM DNA content in 10 nM Tris–Cl, pH8.5, 0.1% Tween20. Libraries were applied to an Illumina flow cell for cluster generation (True Seq SR Cluster Kit V2 cBot) and sequence-by-synthesis single reads of 75 base length using the TruSeq SBS Kit v5 (Illumina) were generated on the Genome Analyzer IIx, using a standard RNA sequencing protocol. Reads were processed using CASAVA (Illumina) to split reads according to adapter indexes and produce fastq files. Read quality was determined by analyzing reads with FastQC.

### NGS data and ingenuity pathway analysis

Reads (~ 10 M per sample) were mapped and gene expression calculated using RSEM software^68^ over mouse assembly GRCm38 with genebuild version 70 from ensembl. Data from RSEM were then analyzed with the Bioconductor package EdgeR^69^ using the generalized linear method. Probes selected by statistical analysis along with their differential expression value were loaded into Ingenuity Pathways Analysis software (IPA) (Ingenuity Systems) for annotation, redundancy checks, network and pathway analysis. Data was processed using IPA to the satisfaction of the right-tailed Fisher's Exact Test, and significant networks based on semantic associations of these genes were generated.

### Immunohistochemical analysis

Mouse hearts and aortas were processed and analyzed as previously described^65^. Tissues were perfused with PBS, removed, fixed in 4% paraformaldehyde for 24 h, incubated for 24 h in PBS supplemented with 30% sucrose, embedded in OCT and cryopreserved at -70 °C. Cryocut cross-sections (5 μm) were then prepared. Samples were blocked for 30 min with 10% horse serum plus 2% BSA (for immunofluorescence) in PBS. Cryocut cross-sections were stained with Oil Red O (0.5% in isopropanol) or Masson’s trichrome stain. Images were acquired using an Olympus BX51 microscope using the fitted 10× or 20× UPlanSApo objectives and Cell Sens Entry Ink acquisition software. Immunofluorescence samples were stained with rat anti-mouse CD68 F4/80 (AbD Serotec, MCA497R) and Cy3-conjugated mouse anti-smooth muscle actin (SMA) (Sigma-Aldrich, C6198). Images were acquired using an Olympus BX51 microscope fitted with 10× or 20× UPlanSApo objectives and Cell Sens Entry Ink acquisition software. The secondary antibody for immunofluorescence was Alexa Fluor 633 goat anti-rat IgG (Invitrogen, A-21094). Nuclei were stained with DAPI. Immunofluorescence images were acquired using an inverted confocal microscope (Carl Zeiss Axio Imager Z2, Apotome.2) fitted with 20× or 40× HCX PL Apo oil immersion objectives and ZEN acquisition software. Images were analyzed using ImageJ (http://rsbweb.nih.gov/ij/index.html) and were processed for presentation with Adobe Photoshop.

### ELISA

Cell culture media from HUVECs were harvested after the indicated treatment. MCP-1 and IL-6 levels were measured by ELISA (BD Bioscience 559017 and 550799) according to the manufacturer’s supplied protocols.

### Adhesion assay under static and flow conditions

THP-1 cells (1–5 × 10^6^) were stained with calcein-AM (Molecular probes) (10 μM) for 30 min at 37 °C after 3 consecutive serum-free RPMI washes. The staining was finished after washing cells in cold RPMI with 10% FBS. THP-1 cells resuspended in RPMI and 10% FBS were added to TNF-α (10 ng/ml) pre-activated HUVEC monolayers, and incubated for 30 min in the incubator. After RPMI repeated washes to remove unbound THP-1 cells the remaining cells were detached with 1 mM EDTA/PBS. Binding was quantified by flow cytometry, and expressed as the ratio of THP-1 (calcein-positive) to HUVEC cells (negative cells). Controls included stained and unstained THP-1 cells, non-labeled HUVECS and HUVECS incubated with the unbound labeled THP-1 cells.

All perfusions under steady flow were performed in µ-Slide VI^0.4^ flow chambers (ibidi, 80601). The human promyelomonocytic cell line THP-1 (ATCC TIB-202) was cultured in RMPI 1640 (Life Technologies11875-093) supplemented with 10% FBS, 100U/mL penicillin and 100 μg/mL streptomycin (Life Technologies 15140-122). THP-1 cells and HUVECs were incubated for 6 h with 2.5 ng/ml TNF-α with or without 1 mM NMP before starting the flow (5.0 dyn/cm^2^). Videos were recorded for 3 min. Peripheral blood mononuclear cells (PBMCs) purchased from Lonza (Cat#CC-2702), were cultured in RPMI 1640 and 15%FBS at 37 °C and 5%CO_2_ before measuring adhesion to an endothelial monolayer under flow conditions. PBMCs (1 × 10^6^ cells/ml) were added to confluent HUVECs which had been pre-stimulated with TNF-α (2.5 ng/ml) for 6 h. HUVECs were also incubated with 1 mM NMP where indicated. Individual 3 min runs of PBMCs were performed at 37 °C at a shear stress of 5.0 dyn/cm^2^. The adhered cells on the endothelial surface were quantified at the end of the perfusion time. The flow chamber was stained for VCAM-1 and CD11b. The shear stress is based on the dynamical viscosity of water at 22 °C, η = 0.01dyn·s/cm^2^, where t is shear stress; f, flow rate; h, dynamical viscosity. In µ-Slide VI^0.4^ flow chambers, the shear stress follows the formula: t[dyn/cm^2^] = 1.316 f[ml/min].

### Statistical analysis

All the experimental outcomes have been analyzed blindly. Mouse experiments were designed to minimize the number of animals needed to give sufficient statistical power. No data were excluded from any analysis. All experiments have been repeated at least twice and with a minimum of two replicates. Statistical analyses were performed with GraphPad Software. Data are expressed as mean ± SEM, and results were analyzed by 1-way ANOVA with Bonferroni post hoc tests or by 2-tailed unpaired Student *t* test. Statistical significance of differences was assigned as follows: * *p* < 0.05, ** *p* < 0.01, *** *p* < 0.001, **** *p* < 0.0001, and ns *p* > 0.05.

## Supplementary information

Supplementary file1 (DOCX 1187 kb)

Supplementary file2 (MOV 22264 kb)

Supplementary file3 (MOV 20868 kb)
