# Supplementary material for: The pharmaceutical solvent N-methyl-2-pyrollidone (NMP) attenuates inflammation through Krüppel-like factor 2 activation to reduce atherogenesis
Source: Sci Rep. 2020 Jul 15;10:11636. doi: 10.1038/s41598-020-68350-2 (PMC7363918; doi:10.1038/s41598-020-68350-2)
Supplement: Supplementary file 1 — Supplementary file1 (DOCX 1187 kb) [file 41598_2020_68350_MOESM1_ESM.docx]

**The pharmaceutical solvent N-methyl-2-pyrollidone (NMP) attenuates inflammation through Krüppel-like factor 2 activation to reduce atherogenesis**

Marta Roche-Molina, Bryn Hardwick, Cristina Sanchez-Ramos, David Sanz-Rosas, Dirk Gewert, Francisco M. Cruz, Andres Gonzalez-Guerra, Vicente Andres, Joaquin A. Palma, Borja Ibañez, Grahame Mckenzie and Juan A. Bernal

**SUPPLEMENTARY FIGURES**

**SUPPLEMENTARY TABLE 1**

| **Gene/Target** | **Primer** | **Sequence (5’-3’)** |
| --- | --- | --- |
| *Klf2* | Fwd  Rev | TTTGCCACTTTCGCCAGC  GGCTCGGGAGGATTCTCG |
| *Gadph* | Fwd  Rev | TTGATGGCAACAATCTCCAC  CGTCCCGTAGACAAAATGGT |
| *18S rRNA* | Fwd  Rev | AACCCGTTGAACCCCATT  CCATCCAATCGGTAGTAGCG |
| *Vcam1* | Fwd  Rev | GGACGATTCCGGCATTTATGTG  AATGACGGTGTCTCCCTCTTTG |
| *Cxcl12* | Fwd  Rev | TGCATCAGTGACGGTAAACCA  TTCTTCAGCCGTGCAACAATC |
| *KLF2* | Fwd  Rev | GCAAGACCTACACCAAGAGTTCG CATGTGCCGTTTCATGTGC |
| *GADPH* | Fwd  Rev | GCCTCCCGCTTCGCTCTCTG  CGTTGACTCCGACCTTCACCTTCC |

**SUPPLEMENTARY TABLE 2**

**Fold Change Log Ratio p-value ID Symbol**

18.614 4.218 1.01E-03 ENSMUSG00000073830 Mup1 (includes others)

15.399 3.945 1.44E-04 ENSMUSG00000029368 ALB

14.539 3.862 7.94E-05 ENSMUSG00000041616 NPPA

14.373 3.845 4.56E-04 ENSMUSG00000005681 APOA2

12.635 3.659 4.65E-11 ENSMUSG00000073876 IL11RA

10.892 3.445 2.50E-04 ENSMUSG00000091345 COL6A5

10.080 3.333 8.90E-08 ENSMUSG00000027832 PTX3

8.769 3.132 1.20E-03 ENSMUSG00000024558 MAPK4

8.639 3.111 9.59E-03 ENSMUSG00000042045 SLN

8.323 3.057 1.70E-02 ENSMUSG00000079015 SERPINA1

7.657 2.937 1.08E-06 ENSMUSG00000095348 LOC102637312

6.811 2.768 3.56E-03 ENSMUSG00000057068 FAM47E

6.751 2.755 1.10E-04 ENSMUSG00000070645 REN

6.728 2.750 5.31E-03 ENSMUSG00000001670 TAT

6.343 2.665 3.45E-03 ENSMUSG00000031344 GABRQ

6.217 2.636 3.90E-03 ENSMUSG00000020469 MYL7

5.774 2.530 2.60E-02 ENSMUSG00000018893 MB

5.475 2.453 2.13E-03 ENSMUSG00000068614 ACTC1

5.395 2.432 5.61E-03 ENSMUSG00000079012 SERPINA3

5.105 2.352 3.30E-02 ENSMUSG00000091898 TNNC1

4.962 2.311 4.24E-14 ENSMUSG00000028989 ANGPTL7

4.846 2.277 6.49E-03 ENSMUSG00000051747 TTN

4.635 2.213 1.77E-02 ENSMUSG00000034755 PCDH11X

4.442 2.151 2.46E-04 ENSMUSG00000047963 STBD1

4.287 2.100 3.88E-06 ENSMUSG00000002565 SCIN

4.143 2.051 3.02E-02 ENSMUSG00000060962 DMKN

3.867 1.951 3.64E-02 ENSMUSG00000035458 TNNI3

3.714 1.893 5.00E-04 ENSMUSG00000031340 Gabre

3.608 1.851 3.22E-02 ENSMUSG00000061086 MYL4

3.481 1.800 1.24E-05 ENSMUSG00000025934 GSTA3

3.435 1.780 5.58E-03 ENSMUSG00000043613 MMP3

3.335 1.738 1.86E-02 ENSMUSG00000031210 Gpr165

3.303 1.724 7.74E-07 ENSMUSG00000033453 ADAMTS15

3.252 1.702 1.72E-02 ENSMUSG00000004791 PGF

3.131 1.646 1.73E-03 ENSMUSG00000074676 FOXS1

3.128 1.645 2.05E-03 ENSMUSG00000049511 HTR1B

3.119 1.641 1.24E-06 ENSMUSG00000055116 ARNTL

3.104 1.634 1.32E-02 ENSMUSG00000085730 Gm3893

3.088 1.627 3.28E-02 ENSMUSG00000003665 HAS1

3.037 1.602 2.17E-03 ENSMUSG00000052374 ACTN2

3.014 1.592 1.84E-02 ENSMUSG00000006014 Prg4

2.958 1.565 8.62E-04 ENSMUSG00000061878 SPHK1

2.953 1.562 4.61E-02 ENSMUSG00000036885 ARHGEF26

2.925 1.548 1.50E-06 ENSMUSG00000023067 CDKN1A

2.923 1.547 3.59E-02 ENSMUSG00000004814 CCL24

2.846 1.509 5.34E-06 ENSMUSG00000020684 RASL10B

2.777 1.474 1.27E-04 ENSMUSG00000031765 Mt1

2.736 1.452 2.81E-04 ENSMUSG00000031762 Mt2

2.731 1.450 2.81E-04 ENSMUSG00000054203 IFI16

2.694 1.430 2.16E-02 ENSMUSG00000027360 HDC

2.636 1.399 7.01E-03 ENSMUSG00000024039 CBS/LOC102724560

2.592 1.374 2.76E-05 ENSMUSG00000003452 BICD1

2.565 1.359 2.61E-02 ENSMUSG00000038583 Pln

2.539 1.344 2.41E-02 ENSMUSG00000053897 SLC39A8

2.486 1.314 2.04E-03 ENSMUSG00000029321 SLC10A6

2.482 1.312 2.00E-02 ENSMUSG00000026070 IL18R1

2.478 1.309 8.62E-04 ENSMUSG00000004951 HSPB1

2.345 1.230 4.54E-04 ENSMUSG00000022853 EHHADH

2.321 1.215 3.50E-03 ENSMUSG00000030089 SLC41A3

2.321 1.215 4.89E-04 ENSMUSG00000030317 TIMP4

2.316 1.212 2.44E-03 ENSMUSG00000024222 FKBP5

2.290 1.195 3.59E-02 ENSMUSG00000019996 MAP7

2.289 1.195 4.17E-03 ENSMUSG00000026414 TNNT2

2.286 1.193 1.58E-02 ENSMUSG00000029641 RASL11A

2.279 1.188 5.41E-04 ENSMUSG00000037362 NOV

2.277 1.187 2.15E-02 ENSMUSG00000030790 ADM

2.246 1.167 3.14E-03 ENSMUSG00000063142 KCNMA1

2.240 1.163 2.48E-02 ENSMUSG00000041949 TANGO6

2.229 1.156 1.10E-04 ENSMUSG00000020122 EGFR

2.203 1.139 1.78E-03 ENSMUSG00000030022 ADAMTS9

2.190 1.131 2.56E-04 ENSMUSG00000032350 GCLC

2.186 1.128 2.09E-03 ENSMUSG00000005514 POR

2.184 1.127 4.37E-02 ENSMUSG00000046694 FAM46B

2.175 1.121 2.81E-03 ENSMUSG00000027074 SLC43A3

2.172 1.119 3.14E-03 ENSMUSG00000044548 DACT1

2.169 1.117 2.81E-04 ENSMUSG00000039701 USP53

2.153 1.106 1.24E-03 ENSMUSG00000078190 Dnm3os

2.143 1.100 1.52E-03 ENSMUSG00000056749 NFIL3

2.135 1.094 3.37E-04 ENSMUSG00000091542 CCL27

2.126 1.088 5.45E-03 ENSMUSG00000020542 MYOCD

2.119 1.083 4.75E-03 ENSMUSG00000036181 HIST1H1C

2.116 1.082 4.46E-03 ENSMUSG00000029821 DFNA5

2.088 1.062 4.76E-03 ENSMUSG00000022094 SLC39A14

2.086 1.060 5.52E-04 ENSMUSG00000028862 MAP3K6

2.070 1.050 2.25E-03 ENSMUSG00000020108 DDIT4

2.069 1.049 2.56E-03 ENSMUSG00000018566 SLC2A4

2.068 1.049 1.45E-03 ENSMUSG00000050666 VSTM4

2.046 1.033 3.17E-02 ENSMUSG00000026077 NPAS2

2.044 1.031 3.94E-03 ENSMUSG00000021750 FAM107A

2.022 1.016 1.39E-02 ENSMUSG00000071005 CCL19

2.009 1.007 1.85E-03 ENSMUSG00000052430 BMPR1B

-2.002 -1.001 2.88E-02 ENSMUSG00000053158 FES

-2.004 -1.003 5.38E-03 ENSMUSG00000032786 ALAS1

-2.005 -1.004 4.77E-03 ENSMUSG00000036622 ATP13A2

-2.013 -1.010 1.12E-02 ENSMUSG00000026582 SELE

-2.018 -1.013 3.27E-02 ENSMUSG00000028599 TNFRSF1B

-2.018 -1.013 7.87E-03 ENSMUSG00000021003 GALC

-2.022 -1.015 1.22E-02 ENSMUSG00000024781 LIPA

-2.023 -1.016 1.64E-02 ENSMUSG00000025804 CCR1

-2.025 -1.018 2.17E-02 ENSMUSG00000022831 HCLS1

-2.027 -1.019 1.63E-02 ENSMUSG00000004207 PSAP

-2.028 -1.020 1.36E-03 ENSMUSG00000032698 LMO2

-2.028 -1.020 1.54E-02 ENSMUSG00000024621 CSF1R

-2.031 -1.022 1.86E-02 ENSMUSG00000034957 CEBPA

-2.031 -1.022 3.71E-03 ENSMUSG00000048758 Rpl29 (includes others)

-2.032 -1.023 6.76E-03 ENSMUSG00000049103 CCR2

-2.039 -1.028 4.79E-02 ENSMUSG00000026832 CYTIP

-2.046 -1.033 2.74E-02 ENSMUSG00000040747 CD53

-2.054 -1.038 3.17E-02 ENSMUSG00000024677 Ms4a6b

-2.069 -1.049 1.28E-02 ENSMUSG00000007891 CTSD

-2.073 -1.052 2.10E-02 ENSMUSG00000024679 MS4A6A

-2.075 -1.053 1.13E-02 ENSMUSG00000031827 COTL1

-2.086 -1.061 5.66E-03 ENSMUSG00000034947 TMEM106A

-2.087 -1.061 4.20E-02 ENSMUSG00000020340 CYFIP2

-2.096 -1.068 1.44E-02 ENSMUSG00000026638 IRF6

-2.097 -1.068 1.87E-02 ENSMUSG00000028312 SMC2

-2.099 -1.070 6.75E-03 ENSMUSG00000003363 PLD3

-2.103 -1.073 5.33E-03 ENSMUSG00000030256 Bhlhe41

-2.103 -1.073 6.06E-03 ENSMUSG00000041362 KIAA1598

-2.105 -1.074 1.61E-02 ENSMUSG00000042129 RASSF4

-2.107 -1.075 4.31E-02 ENSMUSG00000029915 CLEC5A

-2.110 -1.077 1.38E-02 ENSMUSG00000034613 PPM1H

-2.112 -1.079 1.69E-03 ENSMUSG00000073599 ECSCR

-2.115 -1.081 3.38E-02 ENSMUSG00000018774 CD68

-2.121 -1.085 1.28E-02 ENSMUSG00000042228 LYN

-2.127 -1.089 4.09E-03 ENSMUSG00000039062 ANPEP

-2.129 -1.090 1.79E-03 ENSMUSG00000027513 PCK1

-2.132 -1.092 1.39E-02 ENSMUSG00000027322 SIGLEC1

-2.132 -1.092 4.86E-04 ENSMUSG00000022797 TFRC

-2.142 -1.099 6.49E-03 ENSMUSG00000026104 STAT1

-2.143 -1.100 3.50E-03 ENSMUSG00000033685 UCP2

-2.144 -1.101 1.90E-02 ENSMUSG00000030786 ITGAM

-2.144 -1.100 6.35E-03 ENSMUSG00000025969 NRP2

-2.147 -1.102 4.10E-02 ENSMUSG00000030774 PAK1

-2.149 -1.104 9.41E-03 ENSMUSG00000058818 LILRB3

-2.152 -1.106 3.94E-02 ENSMUSG00000038910 PLCL2

-2.154 -1.107 3.46E-02 ENSMUSG00000037443 CEP85

-2.165 -1.114 6.87E-03 ENSMUSG00000026656 FCGR2B

-2.167 -1.116 1.73E-02 ENSMUSG00000058715 FCER1G

-2.171 -1.118 2.90E-03 ENSMUSG00000018008 CYTH4

-2.171 -1.118 3.94E-02 ENSMUSG00000052776 OAS1

-2.173 -1.120 1.16E-02 ENSMUSG00000025355 MMP19

-2.176 -1.122 1.83E-02 ENSMUSG00000001348 Acp5

-2.181 -1.125 3.28E-02 ENSMUSG00000029333 RASGEF1B

-2.182 -1.125 1.07E-02 ENSMUSG00000024965 FERMT3

-2.184 -1.127 1.41E-02 ENSMUSG00000079419 Ms4a6c

-2.187 -1.129 7.03E-03 ENSMUSG00000031266 GLA

-2.188 -1.130 3.94E-02 ENSMUSG00000016496 CD274

-2.193 -1.133 3.54E-02 ENSMUSG00000018654 IKZF1

-2.193 -1.133 6.17E-03 ENSMUSG00000040552 C3AR1

-2.203 -1.139 8.48E-03 ENSMUSG00000026358 RGS1

-2.205 -1.141 7.24E-03 ENSMUSG00000043008 KLHL6

-2.206 -1.142 1.99E-02 ENSMUSG00000038178 SLC43A2

-2.207 -1.142 2.45E-02 ENSMUSG00000028031 DKK2

-2.218 -1.150 1.83E-03 ENSMUSG00000071713 CSF2RB

-2.219 -1.150 7.84E-03 ENSMUSG00000030047 ARHGAP25

-2.219 -1.150 8.44E-04 ENSMUSG00000027962 VCAM1

-2.224 -1.153 1.54E-02 ENSMUSG00000022372 SLA

-2.224 -1.153 2.47E-03 ENSMUSG00000010307 TMEM86A

-2.226 -1.154 1.88E-03 ENSMUSG00000034854 MFSD12

-2.228 -1.156 3.03E-02 ENSMUSG00000087107 AI662270

-2.232 -1.158 1.03E-03 ENSMUSG00000048234 RNF149

-2.233 -1.159 4.38E-03 ENSMUSG00000048865 ARHGAP30

-2.236 -1.161 6.87E-03 ENSMUSG00000026480 NCF2

-2.249 -1.169 1.67E-02 ENSMUSG00000025888 CASP1

-2.249 -1.169 7.99E-03 ENSMUSG00000028874 FGR

-2.250 -1.170 8.15E-03 ENSMUSG00000030579 TYROBP

-2.251 -1.171 1.69E-03 ENSMUSG00000049866 ARL4C

-2.255 -1.173 1.22E-03 ENSMUSG00000041261 CA8

-2.260 -1.176 2.40E-02 ENSMUSG00000031264 BTK

-2.265 -1.179 3.18E-04 ENSMUSG00000021835 BMP4

-2.265 -1.180 6.03E-03 ENSMUSG00000062593 LILRB4

-2.268 -1.182 1.95E-02 ENSMUSG00000045382 CXCR4

-2.269 -1.182 3.21E-04 ENSMUSG00000028957 PER3

-2.274 -1.185 3.10E-02 ENSMUSG00000042684 NPL

-2.276 -1.187 4.31E-02 ENSMUSG00000037731 THEMIS2

-2.277 -1.187 1.26E-02 ENSMUSG00000028581 LAPTM5

-2.279 -1.188 2.12E-02 ENSMUSG00000063060 SOX7

-2.281 -1.190 2.73E-04 ENSMUSG00000022246 RAI14

-2.286 -1.193 3.00E-03 ENSMUSG00000091747 D17H6S56E-5

-2.288 -1.194 4.76E-03 ENSMUSG00000033777 Tlr13

-2.293 -1.197 2.72E-03 ENSMUSG00000036908 UNC93B1

-2.293 -1.197 9.59E-03 ENSMUSG00000033446 LPAR6

-2.297 -1.200 2.15E-02 ENSMUSG00000061132 BLNK

-2.300 -1.201 1.12E-02 ENSMUSG00000026627 TMEM206

-2.302 -1.203 1.06E-02 ENSMUSG00000069792 Wfdc17

-2.306 -1.205 2.05E-02 ENSMUSG00000020437 MYO1G

-2.307 -1.206 3.50E-02 ENSMUSG00000024986 HHEX

-2.308 -1.207 1.63E-02 ENSMUSG00000032076 CADM1

-2.308 -1.206 2.81E-04 ENSMUSG00000064267 HVCN1

-2.312 -1.209 7.87E-03 ENSMUSG00000029816 GPNMB

-2.315 -1.211 2.35E-03 ENSMUSG00000003882 IL7R

-2.322 -1.215 3.28E-02 ENSMUSG00000031004 MKI67

-2.325 -1.217 1.10E-04 ENSMUSG00000007877 TCAP

-2.326 -1.218 4.89E-04 ENSMUSG00000029919 HPGDS

-2.329 -1.220 1.11E-02 ENSMUSG00000030747 DGAT2

-2.329 -1.220 4.10E-02 ENSMUSG00000031558 SLIT2

-2.331 -1.221 1.12E-02 ENSMUSG00000043157 ARL11

-2.335 -1.223 5.01E-03 ENSMUSG00000028327 Stra6l

-2.350 -1.233 5.50E-03 ENSMUSG00000034652 CD300A

-2.354 -1.235 1.27E-03 ENSMUSG00000059498 FCGR2A

-2.355 -1.236 1.01E-02 ENSMUSG00000021423 LY86

-2.356 -1.236 8.68E-03 ENSMUSG00000031438 RNF128

-2.365 -1.242 8.88E-03 ENSMUSG00000045763 BASP1

-2.367 -1.243 5.66E-03 ENSMUSG00000015950 NCF1

-2.368 -1.244 2.81E-03 ENSMUSG00000037902 SIRPA

-2.369 -1.245 1.13E-02 ENSMUSG00000022667 CD200R1

-2.375 -1.248 5.00E-04 ENSMUSG00000017754 PLTP

-2.382 -1.252 4.05E-03 ENSMUSG00000055782 ABCD2

-2.390 -1.257 1.15E-02 ENSMUSG00000033220 RAC2

-2.391 -1.257 1.59E-02 ENSMUSG00000000290 ITGB2

-2.396 -1.261 6.76E-03 ENSMUSG00000032322 PSTPIP1

-2.399 -1.262 2.79E-03 ENSMUSG00000039621 PREX1

-2.403 -1.265 1.01E-03 ENSMUSG00000001128 CFP

-2.404 -1.266 3.85E-03 ENSMUSG00000030789 ITGAX

-2.412 -1.271 2.39E-02 ENSMUSG00000023349 CLEC6A

-2.415 -1.272 1.09E-02 ENSMUSG00000029925 TBXAS1

-2.415 -1.272 1.46E-04 ENSMUSG00000031548 SFRP1

-2.416 -1.272 2.58E-03 ENSMUSG00000036887 C1QA

-2.416 -1.273 9.81E-03 ENSMUSG00000042759 APOBR

-2.419 -1.274 1.13E-03 ENSMUSG00000020143 DOCK2

-2.427 -1.279 5.31E-03 ENSMUSG00000029084 CD38

-2.428 -1.280 2.76E-04 ENSMUSG00000035273 HPSE

-2.428 -1.280 3.99E-03 ENSMUSG00000036469 MARCH1

-2.433 -1.283 2.24E-03 ENSMUSG00000027533 FABP5

-2.436 -1.284 4.61E-03 ENSMUSG00000038147 CD84

-2.437 -1.285 5.36E-03 ENSMUSG00000040522 TLR8

-2.438 -1.286 2.44E-03 ENSMUSG00000021624 CD180

-2.444 -1.289 3.50E-02 ENSMUSG00000028931 KCNAB2

-2.455 -1.296 5.06E-03 ENSMUSG00000045312 LHFPL2

-2.458 -1.297 4.16E-02 ENSMUSG00000045136 TUBB2B

-2.467 -1.303 9.97E-03 ENSMUSG00000038642 CTSS

-2.471 -1.305 2.47E-03 ENSMUSG00000041836 PTPRE

-2.480 -1.311 8.62E-04 ENSMUSG00000024737 SLC15A3

-2.484 -1.313 5.00E-04 ENSMUSG00000026177 SLC11A1

-2.485 -1.313 2.81E-04 ENSMUSG00000025154 ARHGAP19

-2.491 -1.317 2.44E-03 ENSMUSG00000028238 ATP6V0D2

-2.499 -1.321 1.06E-02 ENSMUSG00000026271 GPR35

-2.499 -1.321 7.82E-03 ENSMUSG00000093938 EVI2B

-2.500 -1.322 1.23E-02 ENSMUSG00000054871 TMEM158

-2.501 -1.323 1.01E-03 ENSMUSG00000036905 C1QB

-2.506 -1.325 2.42E-03 ENSMUSG00000021998 LCP1

-2.511 -1.328 1.44E-04 ENSMUSG00000025044 MSR1

-2.521 -1.334 3.20E-02 ENSMUSG00000020709 ADAP2

-2.526 -1.337 1.41E-03 ENSMUSG00000021262 EVL

-2.526 -1.337 5.90E-03 ENSMUSG00000056069 FAM105A

-2.530 -1.339 1.28E-03 ENSMUSG00000024030 ABCG1

-2.541 -1.345 5.15E-03 ENSMUSG00000033192 LPCAT2

-2.548 -1.349 1.55E-02 ENSMUSG00000020399 HAVCR2

-2.549 -1.350 3.46E-04 ENSMUSG00000038623 TM6SF1

-2.550 -1.351 1.80E-02 ENSMUSG00000003644 RPS6KA1

-2.552 -1.352 4.61E-03 ENSMUSG00000046805 MPEG1

-2.557 -1.355 2.73E-04 ENSMUSG00000023034 NR4A1

-2.562 -1.357 1.83E-02 ENSMUSG00000020914 TOP2A

-2.562 -1.357 5.21E-03 ENSMUSG00000000958 SLC7A7

-2.572 -1.363 9.15E-04 ENSMUSG00000025743 SDC3

-2.577 -1.365 9.15E-04 ENSMUSG00000047798 CD300LF

-2.585 -1.370 1.36E-03 ENSMUSG00000051506 WDFY4

-2.586 -1.371 8.62E-04 ENSMUSG00000036896 C1QC

-2.587 -1.371 1.79E-03 ENSMUSG00000040751 LAT2

-2.587 -1.371 4.86E-04 ENSMUSG00000015355 CD48

-2.591 -1.374 8.62E-04 ENSMUSG00000022488 NCKAP1L

-2.601 -1.379 2.86E-03 ENSMUSG00000041515 IRF8

-2.607 -1.382 2.02E-04 ENSMUSG00000046916 MYCT1

-2.608 -1.383 6.96E-03 ENSMUSG00000026979 PSD4

-2.613 -1.386 1.32E-02 ENSMUSG00000020573 PIK3CG

-2.635 -1.398 1.54E-03 ENSMUSG00000004266 PTPN6

-2.642 -1.402 4.69E-03 ENSMUSG00000020865 ABCC3

-2.664 -1.414 1.96E-03 ENSMUSG00000069516 LYZ

-2.667 -1.415 1.40E-02 ENSMUSG00000003283 HCK

-2.675 -1.420 4.54E-04 ENSMUSG00000074785 PLXNC1

-2.676 -1.420 9.42E-03 ENSMUSG00000086825 LOC102641333

-2.679 -1.421 1.10E-04 ENSMUSG00000070348 CCND1

-2.683 -1.424 2.79E-03 ENSMUSG00000025017 PIK3AP1

-2.687 -1.426 4.46E-03 ENSMUSG00000032261 SH3BGRL2

-2.690 -1.428 1.20E-03 ENSMUSG00000000489 PDGFB

-2.691 -1.428 4.98E-02 ENSMUSG00000048924 CCDC125

-2.708 -1.437 1.45E-02 ENSMUSG00000040345 ARHGAP9

-2.718 -1.443 4.38E-03 ENSMUSG00000053835 H2-T24

-2.723 -1.445 1.46E-04 ENSMUSG00000026395 PTPRC

-2.728 -1.448 1.96E-03 ENSMUSG00000044350 LACC1

-2.731 -1.449 9.42E-03 ENSMUSG00000038188 SCARF1

-2.734 -1.451 1.61E-03 ENSMUSG00000063193 CD300LB

-2.735 -1.452 2.56E-03 ENSMUSG00000045362 Tnfrsf26

-2.736 -1.452 3.09E-02 ENSMUSG00000036353 P2RY12

-2.752 -1.461 8.07E-04 ENSMUSG00000034116 VAV1

-2.755 -1.462 4.78E-02 ENSMUSG00000049037 ZNF705A

-2.755 -1.462 4.86E-04 ENSMUSG00000049723 MMP12

-2.756 -1.462 2.81E-04 ENSMUSG00000044811 CD300C

-2.756 -1.463 4.88E-03 ENSMUSG00000023913 PLA2G7

-2.768 -1.469 2.90E-03 ENSMUSG00000022148 FYB

-2.774 -1.472 8.77E-04 ENSMUSG00000078606 Gvin1 (includes others)

-2.783 -1.476 5.31E-04 ENSMUSG00000015947 FCGR1A

-2.790 -1.480 3.12E-04 ENSMUSG00000038811 GNGT2

-2.806 -1.488 8.68E-03 ENSMUSG00000043832 Clec4a3

-2.818 -1.495 5.33E-03 ENSMUSG00000039304 TNFSF10

-2.826 -1.499 5.04E-04 ENSMUSG00000028832 STMN1

-2.842 -1.507 2.30E-05 ENSMUSG00000052160 PLD4

-2.854 -1.513 2.91E-02 ENSMUSG00000052142 RASAL3

-2.886 -1.529 1.31E-04 ENSMUSG00000021451 SEMA4D

-2.895 -1.534 4.54E-04 ENSMUSG00000032089 IL10RA

-2.896 -1.534 5.72E-05 ENSMUSG00000089929 BCL2A1

-2.898 -1.535 9.40E-04 ENSMUSG00000043740 B430306N03Rik

-2.913 -1.542 9.42E-03 ENSMUSG00000037946 FGD3

-2.919 -1.546 9.21E-04 ENSMUSG00000071203 Naip1 (includes others)

-2.927 -1.550 1.53E-03 ENSMUSG00000022415 SYNGR1

-2.934 -1.553 1.83E-02 ENSMUSG00000027737 SLC7A11

-2.955 -1.563 2.27E-05 ENSMUSG00000026981 IL1RN

-2.955 -1.563 7.68E-03 ENSMUSG00000029299 Abcg3

-2.973 -1.572 1.20E-03 ENSMUSG00000054555 ADAM12

-2.998 -1.584 1.91E-05 ENSMUSG00000039004 BMP6

-3.003 -1.586 1.97E-05 ENSMUSG00000024300 MYO1F

-3.009 -1.589 2.70E-03 ENSMUSG00000027199 GATM

-3.017 -1.593 1.20E-03 ENSMUSG00000074151 NLRC5

-3.024 -1.596 5.07E-03 ENSMUSG00000023274 CD4

-3.026 -1.597 3.51E-04 ENSMUSG00000009185 Ccl8

-3.034 -1.601 2.56E-04 ENSMUSG00000031304 IL2RG

-3.037 -1.603 2.01E-05 ENSMUSG00000021822 PLAU

-3.043 -1.606 3.62E-04 ENSMUSG00000055541 LAIR1

-3.045 -1.607 1.63E-02 ENSMUSG00000051735 RINL

-3.062 -1.615 2.35E-02 ENSMUSG00000047810 CCDC88B

-3.092 -1.628 1.02E-03 ENSMUSG00000048163 SELPLG

-3.104 -1.634 2.66E-05 ENSMUSG00000079227 CCR5

-3.111 -1.637 7.71E-03 ENSMUSG00000027399 IL1A

-3.130 -1.646 1.77E-03 ENSMUSG00000039934 GSAP

-3.130 -1.646 3.66E-02 ENSMUSG00000070691 RUNX3

-3.154 -1.657 1.43E-02 ENSMUSG00000071068 TREML2

-3.194 -1.675 2.16E-03 ENSMUSG00000022439 PARVG

-3.205 -1.680 2.00E-07 ENSMUSG00000037411 SERPINE1

-3.205 -1.680 9.06E-06 ENSMUSG00000025877 HK3

-3.225 -1.689 1.25E-07 ENSMUSG00000021367 EDN1

-3.229 -1.691 5.34E-06 ENSMUSG00000018920 CXCL16

-3.249 -1.700 2.19E-05 ENSMUSG00000068196 COL8A1

-3.254 -1.702 2.20E-04 ENSMUSG00000079547 HLA-DMB

-3.290 -1.718 1.98E-02 ENSMUSG00000002668 DENND1C

-3.290 -1.718 5.79E-06 ENSMUSG00000079293 CLEC7A

-3.315 -1.729 2.15E-03 ENSMUSG00000047180 NEURL3

-3.316 -1.729 2.02E-04 ENSMUSG00000044258 Ctla2a/Ctla2b

-3.317 -1.730 8.92E-04 ENSMUSG00000060550 HLA-A

-3.323 -1.733 2.67E-02 ENSMUSG00000024675 Ms4a4b

-3.324 -1.733 2.05E-03 ENSMUSG00000044827 TLR1

-3.341 -1.740 5.45E-08 ENSMUSG00000032487 PTGS2

-3.363 -1.750 2.30E-04 ENSMUSG00000057191 C19orf38

-3.373 -1.754 2.78E-02 ENSMUSG00000026228 HTR2B

-3.387 -1.760 4.89E-04 ENSMUSG00000015340 CYBB

-3.410 -1.770 3.16E-02 ENSMUSG00000018983 E2F2

-3.411 -1.770 1.20E-03 ENSMUSG00000037649 HLA-DMA

-3.427 -1.777 3.53E-02 ENSMUSG00000073854 AI427809

-3.451 -1.787 2.47E-03 ENSMUSG00000062380 TUBB3

-3.464 -1.792 1.02E-04 ENSMUSG00000038179 SLAMF7

-3.469 -1.795 1.07E-04 ENSMUSG00000053063 CLEC12A

-3.472 -1.796 4.01E-05 ENSMUSG00000004707 LY9

-3.477 -1.798 7.53E-09 ENSMUSG00000054690 EMCN

-3.491 -1.804 2.00E-07 ENSMUSG00000024672 MS4A7

-3.492 -1.804 7.94E-05 ENSMUSG00000078945 NAIP

-3.493 -1.805 8.88E-04 ENSMUSG00000021886 GPR65

-3.511 -1.812 2.81E-04 ENSMUSG00000000682 Cd52

-3.531 -1.820 5.45E-08 ENSMUSG00000026628 ATF3

-3.535 -1.822 5.58E-03 ENSMUSG00000047592 Nxpe5

-3.556 -1.830 1.01E-03 ENSMUSG00000030214 PLBD1

-3.612 -1.853 2.24E-03 ENSMUSG00000022534 MEFV

-3.621 -1.857 1.97E-02 ENSMUSG00000017697 ADA

-3.631 -1.860 9.87E-03 ENSMUSG00000024013 FGD2

-3.651 -1.868 4.16E-02 ENSMUSG00000031530 DUSP4

-3.655 -1.870 2.60E-02 ENSMUSG00000027347 RASGRP1

-3.685 -1.882 9.10E-06 ENSMUSG00000040152 THBS1

-3.691 -1.884 3.03E-03 ENSMUSG00000031389 ARHGAP4

-3.726 -1.898 5.31E-05 ENSMUSG00000004730 EMR1

-3.790 -1.922 2.08E-02 ENSMUSG00000043939 9830107B12Rik

-3.811 -1.930 8.30E-05 ENSMUSG00000059089 FCGR3A/FCGR3B

-3.852 -1.945 3.22E-02 ENSMUSG00000037010 APLN

-3.874 -1.954 9.42E-03 ENSMUSG00000001228 UHRF1

-3.890 -1.960 1.32E-02 ENSMUSG00000036526 CARD11

-3.890 -1.960 1.63E-02 ENSMUSG00000057135 SCIMP

-3.897 -1.962 3.60E-02 ENSMUSG00000079685 ULBP1

-3.912 -1.968 4.65E-02 ENSMUSG00000087691 LOC102640368

-3.934 -1.976 2.88E-10 ENSMUSG00000028195 CYR61

-3.943 -1.979 2.60E-02 ENSMUSG00000030187 Klra2

-3.977 -1.992 1.52E-03 ENSMUSG00000028459 CD72

-3.986 -1.995 8.62E-04 ENSMUSG00000026494 KIF26B

-3.990 -1.997 2.05E-02 ENSMUSG00000078922 Tgtp1/Tgtp2

-3.993 -1.997 1.61E-03 ENSMUSG00000050578 MMP13

-3.998 -1.999 5.45E-08 ENSMUSG00000028262 Clca1/Clca2

-4.003 -2.001 1.46E-02 ENSMUSG00000096334 SH2D1B

-4.021 -2.008 1.61E-03 ENSMUSG00000051457 SPN

-4.036 -2.013 3.14E-03 ENSMUSG00000033213 C15orf48

-4.078 -2.028 2.84E-02 ENSMUSG00000030149 Klrk1

-4.087 -2.031 4.90E-02 ENSMUSG00000097194 9330175E14Rik

-4.101 -2.036 7.24E-03 ENSMUSG00000054510 Gm14461

-4.122 -2.043 2.81E-03 ENSMUSG00000074480 MEX3A

-4.141 -2.050 1.53E-02 ENSMUSG00000000409 LCK

-4.141 -2.050 9.32E-04 ENSMUSG00000024397 AIF1

-4.142 -2.050 8.14E-03 ENSMUSG00000040204 KIAA0101

-4.145 -2.052 8.48E-03 ENSMUSG00000046245 PILRA

-4.146 -2.052 2.68E-03 ENSMUSG00000075010 AW112010

-4.182 -2.064 6.42E-06 ENSMUSG00000070427 IL18BP

-4.255 -2.089 2.29E-04 ENSMUSG00000040061 PLCB2

-4.262 -2.092 1.27E-02 ENSMUSG00000039193 NLRC4

-4.262 -2.092 3.72E-02 ENSMUSG00000005763 CD247

-4.300 -2.104 8.30E-05 ENSMUSG00000036594 HLA-DQA1

-4.329 -2.114 7.22E-07 ENSMUSG00000030577 CD22

-4.333 -2.115 6.50E-03 ENSMUSG00000030745 IL21R

-4.363 -2.125 1.05E-04 ENSMUSG00000024610 CD74

-4.400 -2.137 8.98E-05 ENSMUSG00000040829 ZMYND15

-4.406 -2.140 1.47E-04 ENSMUSG00000021175 CDCA7L

-4.443 -2.152 2.86E-02 ENSMUSG00000089722 CD300LD

-4.485 -2.165 2.68E-03 ENSMUSG00000046275 TUSC5

-4.530 -2.179 8.30E-05 ENSMUSG00000073421 HLA-DQB1

-4.610 -2.205 7.65E-06 ENSMUSG00000036641 CCDC148

-4.654 -2.218 2.00E-07 ENSMUSG00000025993 SLC40A1

-4.677 -2.226 1.10E-04 ENSMUSG00000030830 ITGAL

-4.677 -2.226 4.70E-05 ENSMUSG00000060586 HLA-DRB5

-4.703 -2.234 3.38E-04 ENSMUSG00000021322 AOAH

-4.736 -2.244 7.60E-03 ENSMUSG00000073412 Lst1

-4.792 -2.261 3.90E-03 ENSMUSG00000051279 GDF6

-4.940 -2.304 1.47E-04 ENSMUSG00000053318 SLAMF8

-5.002 -2.322 8.30E-05 ENSMUSG00000030159 CLEC1B

-5.025 -2.329 5.91E-04 ENSMUSG00000051048 P4HA3

-5.062 -2.340 4.53E-02 ENSMUSG00000076609 IGKC

-5.076 -2.344 1.28E-03 ENSMUSG00000070390 NLRP1

-5.176 -2.372 1.70E-02 ENSMUSG00000022126 IRG1

-5.179 -2.373 1.76E-02 ENSMUSG00000015879 Fam184b

-5.212 -2.382 4.65E-11 ENSMUSG00000059824 DBP

-5.306 -2.408 1.61E-03 ENSMUSG00000050075 GPR171

-5.311 -2.409 6.28E-06 ENSMUSG00000036853 MCOLN3

-5.333 -2.415 1.97E-03 ENSMUSG00000052353 CEMIP

-5.346 -2.418 1.10E-03 ENSMUSG00000035459 STAB2

-5.530 -2.467 1.61E-03 ENSMUSG00000045322 TLR9

-5.580 -2.480 6.55E-06 ENSMUSG00000029581 FSCN1

-5.808 -2.538 7.24E-03 ENSMUSG00000054641 MMRN1

-5.823 -2.542 8.62E-04 ENSMUSG00000030117 GDF3

-5.879 -2.556 7.65E-06 ENSMUSG00000052336 CX3CR1

-6.005 -2.586 4.42E-11 ENSMUSG00000015854 CD5L

-6.065 -2.601 3.14E-03 ENSMUSG00000053977 CD8A

-6.336 -2.663 2.34E-03 ENSMUSG00000020826 NOS2

-6.417 -2.682 1.20E-03 ENSMUSG00000070873 LILRA5

-6.707 -2.746 1.02E-04 ENSMUSG00000009292 TRPM2

-6.734 -2.751 3.85E-08 ENSMUSG00000062082 CD200R1L

-7.074 -2.823 4.30E-07 ENSMUSG00000030162 OLR1

-7.107 -2.829 8.47E-10 ENSMUSG00000028194 DDAH1

-7.321 -2.872 1.15E-03 ENSMUSG00000044349 Snhg11

-7.773 -2.958 8.84E-06 ENSMUSG00000022504 CIITA

-9.524 -3.252 4.34E-08 ENSMUSG00000034438 Gbp8

-14.215 -3.829 1.86E-05 ENSMUSG00000095079 IGHA1

-14.229 -3.831 2.52E-11 ENSMUSG00000054588 GBP6

-20.493 -4.357 3.75E-05 ENSMUSG00000016283 H2-M2

-46.885 -5.551 2.16E-09 ENSMUSG00000022483 COL2A1

-705.007 -9.461 2.82E-16 ENSMUSG00000089739 TMEM189
